# Characterization of Sialic Acid Affinity of the Binding Domain of Mistletoe Lectin Isoform One

**DOI:** 10.3390/ijms22158284

**Published:** 2021-07-31

**Authors:** Soran Mohammed, Natalie Ferry

**Affiliations:** 1 Biomedical Research Centre, School of Environment & Life Sciences, Peel Building, University of Salford, Salford M5 4NT, UK; 2 Department of Biotechnology and Crop Science, University of Sulaimani, Sulaimani 46001, Iraq

**Keywords:** lectin, sialic acid, glycan binding protein

## Abstract

Sialic acid (Sia) is considered as one of the most important biomolecules of life since its derivatives and terminal orientations on cell membranes and macromolecules play a major role in many biological and pathological processes. To date, there is only a limited number of active molecules that can selectively bind to Sia and this limitation has made the study of this glycan challenging. The lectin superfamily is a well-known family of glycan binding proteins, which encompasses many strong glycan binding peptides with diverse glycan affinities. Mistletoe lectin (ML) is considered one of the most active members of lectin family which was initially classified in early studies as a galactose binding lectin; more recent studies have suggested that the peptide can also actively bind to Sia. However, the details with respect to Sia binding of ML and the domain responsible for this binding are left unanswered because no comprehensive studies have been instigated. In this study, we sought to identify the binding domain responsible for the sialic acid affinity of mistletoe lectin isoform I (MLI) in comparison to the binding activity of elderberry lectin isoform I (SNA), which has long been identified as a potent Sia binding lectin. In order to execute this, we performed computational carbohydrate-protein docking for MLB and SNA with Neu5Ac and β-Galactose. We further analyzed the coding sequence of both lectins and identified their glycan binding domains, which were later cloned upstream and downstream to green fluorescent protein (GFP) and expressed in *Escherichia coli* (*E. coli*). Finally, the glycan affinity of the expressed fusion proteins was assessed by using different biochemical and cell-based assays and the Sia binding domains were identified.

## 1. Introduction

The plant kingdom is a rich of source of glycan binding peptides with vast diversity spread throughout the kingdom and the occurrence of a specific peptide is not often restricted to a specific plant family. Lectins are one of the most abundant family of glycan binding peptides observed in almost all plant families which can actively bind to carbohydrates through different types of non-covalent interactions, such as hydrophobic interactions, hydrogen bonds and metal coordination. Furthermore, the existence of a large number of hydroxyl groups on the surface of glycans makes them an appropriate binding partner for lectins, especially in the complex networks of hydrogen bonds where the hydroxyl groups function as both donor and acceptor [1,2].

Whilst the multivalent nature of lectins probably provides them with ultimate selectivity, their specificity to a primary monosaccharide is a major factor in their carbohydrate recognition and it is usually an indicator for a particular branched carbohydrate to be recognized by a specific lectin. Thus, complex carbohydrates bearing glucose (Glc) or mannose (Man) are bound by the Glc/Man-specific lectins (e.g., *Lathyrus ockrus* and Con A) but galactose (Gal)-specific lectins (e.g., *Erythrina corallodendron*, EcorL) require the presence of Gal to perform this binding [3,4]. Lectins can also agglutinate cells because of their divalent or oligovalent structures, which provides each lectin molecule at least two glycan binding sites. This provides the lectin molecules with an ability to bind to membrane polysaccharides and glycoproteins and helps cross-linking between cells via their cell surface carbohydrates or between other molecules bearing carbohydrates [5].

Mistletoe lectin (ML) from (*Viscum album* L.) is a non-leguminous lectin composed of three different isoforms: MLI (viscumin), MLII and MLIII. Each isoform is composed of a toxic chain and a glycan binding chain [6]. Despite the fact that these three isoforms have very similar primary coding sequences, they possess different carbohydrate binding affinities. Isoform one (MLI) is known to possess affinity toward D-galactose, which is 120-fold stronger than its affinity to N-acetylgalactosamine (GalNAc) [7]. The second isoform (MLII) has equivalent affinity to both D-galactose and GalNAc, whereas the third (MLIII) isoform has affinity only to GalNAc. These three isoforms are also different in terms ofmolecular weight as both the toxic chain (A) and the binding chain (B) of MLI (A: 29 kDA, B: 34 kDA) are larger than the chains of MLII and MLIII (A: 27 kDA, B: 32 kDA) (A: 25 kDA, B: 30 kDA), respectively [7,8,9].

The main and major isoform of mistletoe lectin is isoform one (MLI), which provides immunomodulatory potency to the lectin and it is a type II ribosome-inactivating protein (RIPII). The A-subunit of MLI is a toxic protein domain and contains a highly specific N-glycosidase which can effectively halt cellular protein synthesis via modifying the 28S rRNA of the eukaryotic ribosome 60S subunit. The B-subunit of MLI is a glycan binding domain that also increases the cytotoxicity of the toxic subunit by causing cell agglutination and facilitation of cell internalization [9,10].

Sialic acid is the most common type of terminal groups of vertebrate carbohydrates with a surprising taxonomic distribution found in both the deuterostome lineage animals and some of their pathogens [11,12]. The two main types of sialic acid are N-glycolylneuraminic acid (Nau5Gc) and N-acetylneuraminic acid (Nau5Ac), as the former (Nau5Gc) comes from the latter (Nau5Ac) after hydroxylation of its acetyl group by CAMP-Nau5Ac hydroxylase (CMAH). Mammalian cells possess excessive amounts of sialic acid on their membrane pericellular matrix (glycocalyx) and this sialylation pattern on cell membranes changes in diseased cells, for example, the N-glycans of the oligosaccharides on the membrane of melanoma cancer cells were analyzed by Hoja-Łukowicz and colleagues at different progression stages (primary and metastasis). The cells were found highly and abnormally sialilated with an abundant amount of α2-6-linked sialic acids, especially on the L1CAM oligosaccharides on the outer membrane of metastatic melanoma cells [13,14].

The experimental identification of the structural binding sites between glycan binding proteins (GBP) and glycans requires three-dimensional structures (3D) of both parts, which is not easy to accomplish with crystallography due to various issues such as glycans intrinsic resistance to crystallization. The development of advanced tools to theoretically predict the structure of glycans has facilitated the augmentation of experimental data and conquered some of these limitations [15]. The study of receptor recognition by GBPs has also widely expanded and extended into various fields ranging from biology [16] to medicine [17,18] and biomass polysaccharide recognition [19]. The biggest step forward in determining GBP specificity was the introduction of glycan array screening, which allowed researchers to determine various glycans that can be recognized by a particular GBP [20]. However, despite providing a significant dataset screening such as this, there are still limitations, particularly, in terms of presentation of the ligand on the array [21]. Thus, the combination of both computational and laboratory experimental screening as presented here could be an ideal option to further explore the glycan affinity of different GBPs.

## 2. Results

### 2.1. Protein Sequence Analysis

**Figure 1:**
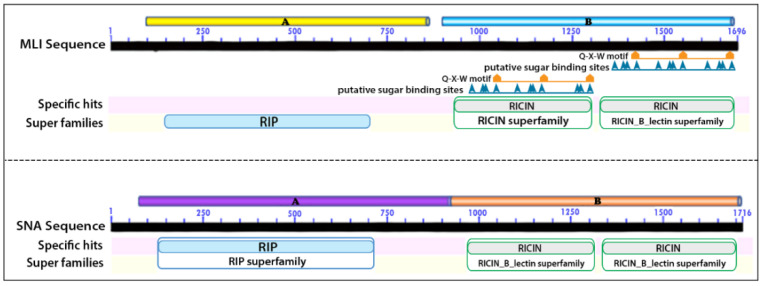
Analysis map of MLI and SNA coding sequences.

The alignment of full sequences of MLI and SNA against the sequences of previously known glycan binding lectins helped to identify the exact position of the main toxic A and glycan binding B domains of MLI and SNA. Numbers of glycan binding subdomains were also identified among the main glycan binding domains as shown in Figure 1.

### 2.2. Virtual Identification of the Glycan Binding Pockets

**Figure 2:**
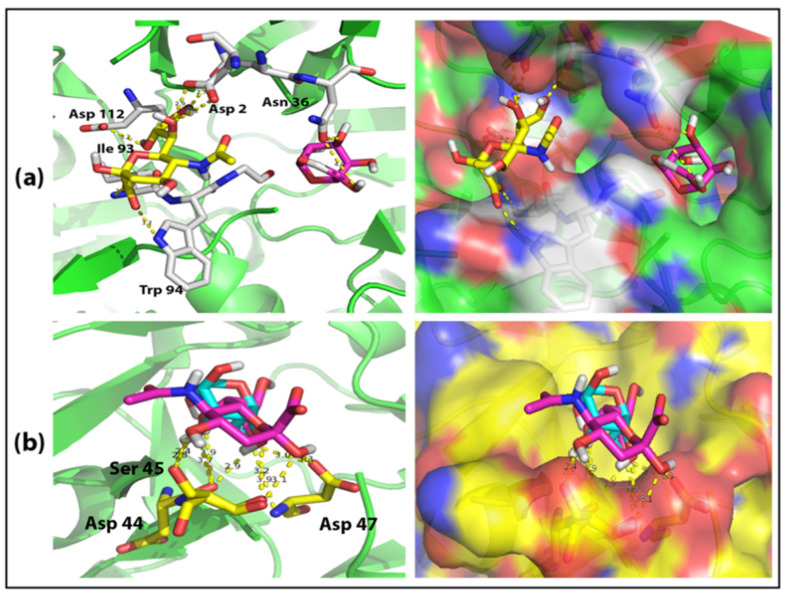
Binding poses of virtual docking. (**a**) MLB + Neu5Ac and β-Galactose; (**b**) SNA-B + Neu5Ac and β-Galactose.

The virtual Glycan-GBP docking was used as a first step to identify the potential Neu5Ac binding pocket on MLB. In order to conduct this, the structure of both Neu5Ac and β-Galactose (Gal) was docked against the binding domain of both MLI (MLB) and SNA (SNA-B). The detected binding poses with the highest affinity of 5.0 and 5.4 kcal/mol in the docking of MLB with Neu5Ac and Gal, respectively, showed that both glycans bind to the same pocket on MLB but with a short distance between their binding sites (Figure 2a). Several binding interactions were detected between MLB and Neu5Ac as follows: the oxygen atoms on the carboxyl group of Neu5AC were observed to interact with the backbone NH of Ile 93 and side chain indole NH of Trp 94 through an ionic bond; on the other side of the glycan, the O7 and O8 atoms H-bonded to the side chain COO of Asp 2 and Asp 211, respectively; the O9 atoms H-bonded to the side chain carbonyl of Asn 35. However, the binding of Gal to MLB appeared only through H-bonding of O3 and O6 atoms to the side chain carbonyl of Asn 36.

The binding of SNA to Neu5Ac and β-Galactose was observed to be different than the binding of MLB as both glycans attached to the exact site on the exact pocket of SNA with the affinity of 6.2 and 5.8 kcal/mol for SNA + Neu5Ac and SNA + Gal, respectively (Figure 2b). The binding of both glycans was found to be solely though H-bonding to Asp 44, Ser 45 and Asp 47. The carboxylic group of Asp 44 was found H-bonded to O5 and O9 atoms of Neu5Ac and O3 and O4 atoms of Gal. The side chain OH of Ser 45 was found H-bonded to O4 and O3 atoms of Neu5Ac and Gal, respectively, but its backbone CO was found H-bonded to O2 and O4 atoms of Neu5Ac and Gal, respectively. Finally, the backbone NH of Asp 47 was found H-bonded to O9 atom of Neu5Ac but its backbone CO was found H-bonded to O6 atom of Gal.

These results suggest that the sialic acid and galactose binding sites on MLB are located on two different areas of the same pocket, which allows the protein to bind to both glycans simultaneously. However, the binding of both glycans to the same site of the same pocket on SNA-B suggests that the protein has ability to bind to both glycans but the higher affinity of SNA toward Neu5Ac may increase the chance of binding with Nue5Ac more than galactose.

### 2.3. PCR Amplification of MLA and MLB

**Figure 3:**
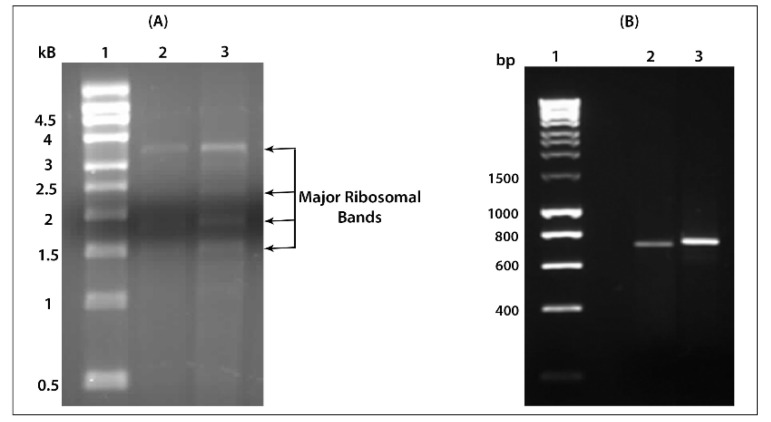
(**A**) Formaldehyde electrophoreses gel (1%). Lane 1: RNA size marker; Lane 2 and 3: two different total RNA samples from mistletoe leaves. (**B**) Agarose gel (1%) showing amplified chain A and chain B of mistletoe lectin. Lane 1: HyperLadder I; Lane 2: amplified chain A (MLA); Lane 3: amplified chain B (MLB).

The sequences of the toxic chain (MLA) and the binding chain (MLB) of mistletoe lectin, 762 bp and 789 bp, respectively, were successfully amplified using cDNA templates reverse transcribed from the total RNA of European mistletoe. The extracted RNA was visualized on a formaldehyde electrophoreses gel, as shown in Figure 3A, and the size of the amplified chains is confirmed by electrophoresis agarose gel as shown in Figure 3B.

Several sequencing reactions were also performed on three biological replicates of the amplified products to eliminate the possible experimental false mismatch. We found that the coding sequences of both chains were slightly different than compared to the query sequence on NCBI GeneBank (AY377890.1) and some base mismatches were detected in both MLA and MLB chains. The result of the alignment of amplified MLA and MLB sequences with the query sequences showed that MLA had seven amino acids mutated as follows: ATA (Isoleucine), GTT (Valine), ATG (Methionine) and CAG (Glutamine) were replaced with TTA (Leucine), ATT (Isoleucine), GTG (Valine) and CAT (Histidine), respectively (Figure S1 in Supplementary Data ). Moreover, MLB had three amino acids mutated as follow: GAA (Glutamine acid), ATC (Isoleucine) and GTG (Valine) were replaced with GGA (Glycine), GTC (Valine) and GCG (Alanine), respectively (Figure S2 in Supplementary Data).

We believe that the main reason behind those mutations was the origin of the mistletoe plants, which were from the United Kingdom and Ukraine for our sequence and the query sequence, respectively.

### 2.4. Lectin-GFP Fusion Proteins

**Figure 4:**
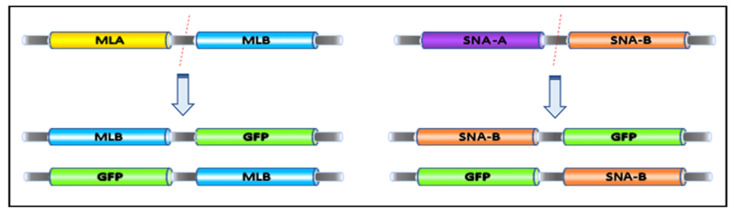
Schematic diagram of MLB-GFP and SNA-B-GFP fusion constructs.

Since the sialic acid binding activity of the binding domain of mistletoe lectin isoform I (MLB) alone and without the toxic domain (MLA) had never been examined separately, we N/C-terminally fused the MLB sequence to green florescent protein (GFP) in order to generate two recombinant fusion proteins for subsequent sialic acid binding characterization. The same fusion proteins were also generated for the binding domain (SNA-B) of elderberry lectin (*Sambucus nigra*), which is a well-known sialic acid binding lectin and used as a positive control (Figure 4). All the fusion proteins were successfully expressed in *E. coli*.

**Figure 5:**
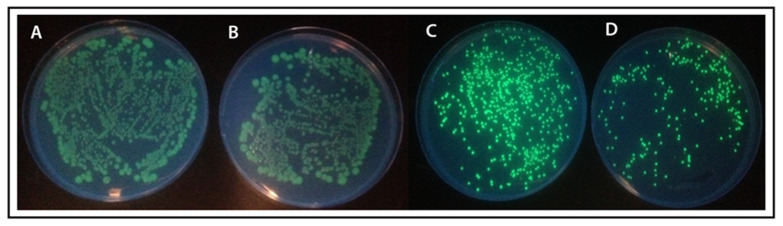
On plate protein expresssion screening. Plate (**A**,**B**): *N*-terminally fused MLB and SNA to GFP; Plate (**C**,**D**): *C*-terminally fused MLB and SNA to GFP.

On-plate screening of the fusion proteins was performed to assess the bacterial expression level of the fusion proteins. The results showed that the fusion proteins made from *C*-terminally fused MLB and SNA-B to GFP were expressed better as they showed higher GFP emission than the *N*-terminally fused MLB and SNA-B to GFP (Figure 5). This might be because of the better folding of the proteins or less protein–protein interaction between GFP and the lectin domains in the *C*-terminal cloning orientation. Therefore, the *C*-terminally fused recombinant proteins were selected for the large-scale protein expression.

### 2.5. Affinity Protein Purification

**Figure 6:**
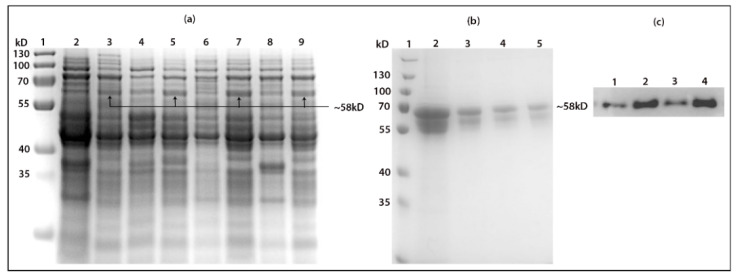
(**a**) SDS page of expressed *N*-terminally and *C*-terminally fused MLB and SNA-B to GFP. Lane 2, 4, 6 and 8 are non-induced protein samples: Lane 3, 5, 7 and 9 are induced protein samples from GFP + MLB, MLB + GFP, GFP + SNA and SNA + GFP fusion proteins, respectively. (**b**,**c**) SDS page of purifird GFP + MLB, MLB + GFP, GFP + SNA and SNA + GFP, respectively.

The purpose of using affinity purification chromatography was to purify the recombinant fusion proteins from the total cell lysate as well as the instant assessment of the sialic acid binding of the fusion proteins by assessing their binding to the free sialic acid residues on the fetuin glycoprotein of the column. The fractions of the purified proteins were analyzed with SDS-PAGE and immunoblotting and showed that both *N*-erminus and *C*-terminus fusion proteins successfully bound to the sialic acid residues. However, a higher amount of purified protein was recovered from the elution steps of *C*-terminally fused recombinant proteins of both MLB and SNA. This indicates that the sialic acid binding of *C*-terminally fused proteins was tighter than the *N*-terminally fused protein as both proteins were expressed and purified in identical conditions. This was also compatible with our previous finding of the on-plate screening of the fusion proteins (Figure 6).

### 2.6. Erythrocytes Hemagglutination by MLB-GFP and SNA-GFP

The minimum agglutination concentrations (MAC) of MLB-GFP and SNA-GFP fusion proteins to agglutinate erythrocytes were identified by a hemagglutination assay using 1% sheep erythrocytes. Both fusion proteins agglutinated the erythrocytes at a MAC of 0.3 μM and 0.25 μM, respectively. This shows that MLB alone without the toxic domain retained the native hemagglutination activity similar to the full-length counterpart lectins.

**Figure 7:**
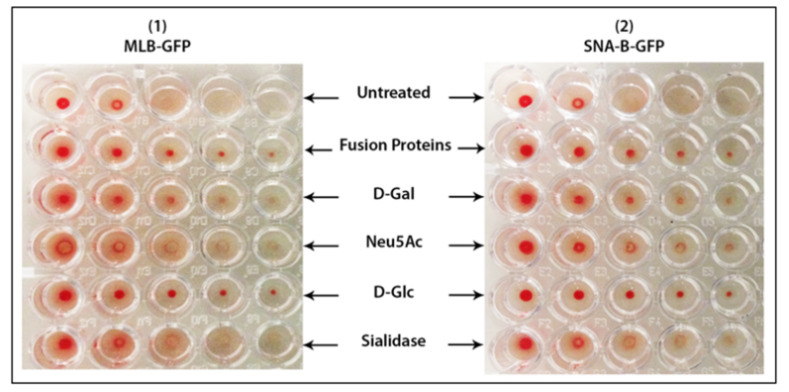
Hemagglutination assay using sheep erythrocytes. First row, untreated erythrocytes; second row, erythrocytes dosed with untreated MLB-GFP (**1**) and SNA-B-GFP (**2**) fusion proteins; third row, erythrocytes dosed with MLB-GFP + Sialic acid (**1**) and SNA-B-GFP + Sialic acid (**2**); fourth row, erythrocytes dosed with MLB-GFP + Galactose (**1**) and SNA-B-GFP + Galactose (**2**); fifth row, erythrocytes dosed with MLB-GFP + Glucose (**1**) and SNA-B-GFP + Glucose (**2**); sixth row, sialidase treated erythrocytes dosed with MLB-GFP (**1**) and SNA-B-GFP (**2**) fusion proteins.

Both identified MAC of MLB-GFP and SNA-GFP were then used in the hemagglutination inhibition assays using a serial dilution of 1% erythrocytes and different hemagglutination inhibition agents such as D-Gal, Neu5Ac, D-Glc and sialidase enzyme. The fusion proteins were observed to actively agglutinate the erythrocytes when they were used alone; however, their hemagglutination activity was partially compromised when they were pre-incubated with D-Gal, especially MLB-GFP, which showed slightly less activity than SNI-B-GFP (Figure 7). Moreover, both MLB-GFP and SNA-GFP were observed to lose their hemagglutination activity when they were pre-incubated with Neu5Ac and the main reason could be the pre-occupation of their Sia binding sites by Neu5Ac, which prevented them from binding to the terminal Sia on the membrane of erythrocytes and subsequently caused reduced agglutination. A similar result was also detected in the case of sialidase treated erythrocytes because the cells did not possess terminal Sia on their membrane glycoproteins anymore and this stopped them from being bound and agglutinated by MLB-GFP and SNA-GFP. These results suggest that MLB can bind to sialic acid similar to SNA. On the other hand, the incubation of both fusion proteins with D-Glc had no impact on their agglutination activity; this agrees with the previous findings that both fusion proteins have zero affinity for glucose (Figure 7).

### 2.7. MLB-GFP and SNA-GFP Binding to Mammalian Cells

The overexpression of α2, 6 sialic acid on the membrane of WM-266-4 metastatic melanoma cells was previously confirmed in a study conducted by Hoja-Łukowicz and Link-Lenczowsk, 2012. Therefore, we believed that WM-266-4 cell line is a suitable candidate for mammalian cell-based activity assessment of the fusion proteins.

**Figure 8:**
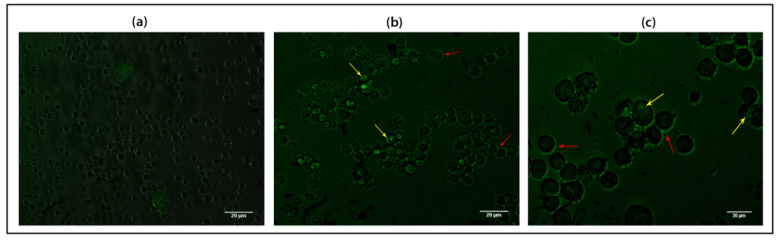
Metastatic melanoma cells (WM-266-4) incubated with 0.3 µM of (**a**) GFP; (**b**) MLB + GFP and (**c**) SNA + GFP for 24 h and screened under luminescence imaging microscopy. The RED arrows show the attachment of the fusion proteins to the cell membranes and cell agglutination. The YELLOW arrows show cell internalization of the fusion proteins.

The epifluorescence microscopy screening of WM-266-4 cells treated with MLB-GFP and SNA-GFP showed that both fusion proteins successfully attached to the membrane of the cells and were successfully internalized across their membranes as the GFP florescence was detected on the outer surface of the cell membranes as well as inside the cells (Figure 8). The cells were also observed to be agglutinated by both fusion proteins, which confirms their agglutination activity and this is compatible with the outcome of our hemagglutination inhibition assay.

## 3. Discussion

The vast diversity of sialic acid distribution among different organisms and its crucial biological functions render this glycan an important biomolecule to investigate more extensively, while understanding the biofunctions of this glycan and its derivatives is still relatively limited. In order to further study the biological functions of Sia, selective and efficient sialic acid binding molecules need to be identified in order to be able to comprehensively study the undiscovered functions of the glycan.

The contradictory outcomes of previous studies regarding the glycan affinity of mistletoe lectin left the affinity of this lectin as an answered question, particularly since older studies classified the lectin as a galactose binding while more recent studies detected sialic acid binding activity from an isoform of the lectin. Furthermore, the concept of Sia binding of mistletoe lectin remained vague for a long time because only limited experiments were conducted and they only focused on sialic acid binding activity of the total MLI isolated from extract of mistletoe [22]. Therefore, we used a genetic fusion approach to precisely examine the Sia affinity of the binding domain (MLB) of mistletoe lectin isoform I (MLI) without the toxic MLA domain in this study. The first binding assay conducted in the affinity purification step utilized the terminal sialic acids on the surface of fetuin glycoproteins and we discovered that MLB can actively bind to Sia as observed for SNA, which is a well-known sialic acid binding lectin. This strongly suggests that the classification of mistletoe lectin as a galactose binding lectin was purely due to a lack of experimentation.

Subsequently, hemagglutination and inhibition assays were necessary for comparing the Sia binding of MLB with galactose binding. The slight inhibition of the erythrocyte agglutination by galactose treated MLB-GFP confirmed that MLB has the affinity to galactose as well. This is compatible with the previously reported galactose affinity of mistletoe lectin [23]. However, the agglutination inhibition caused by galactose was not as strong as the inhibition caused by the Sia treated MLB-GFP and this provides a clear indication that MLB binds to the terminal Sia on erythrocytes stronger than binding to galactose. Moreover, the binding of both MLB-GFP and SNA-GFP to the terminal Sia on the membrane glycoproteins of WM-266-4 human melanoma cells confirmed the fact that both lectin domains can similarly bind to the Sia rich glycoproteins on the membrane of mammalian cells. Furthermore, both binding domains preserved their agglutination capacity, which is a well-known biological function possessed by their native full-length counterpart lectins and a crucial function for facilitating their cell membrane binding and cell internalization. The observed activities of mistletoe lectin treatment on human melanoma cells are in line with results of a study conducted by Hoja et al. 1995, which investigated the impact of mistletoe lectin treatment on the interleukin-12 (IL-12) secretion in human peripheral blood mononuclear cells and the treatment was observed to enhance the secretion of an active form of IL-12.

The results of our computational screening and the assays conducted in this study confirm that the binding chain (MLB) of mistletoe lectin isoform I (MLI) can actively bind to sialic acid and that the glycan binding activity of both MLB and SNA alone and without their toxic domains remains intact. Therefore, both domains could be used as potent binding agents or monobody to specifically target sialic acid in different biological systems. This could eventually permit the use of the domains in biomedical studies as diagnostic tools or potentially as therapeutic agents in order to activate certain biological signals via binding to sialic acid on specific counter-receptors.

## 4. Materials and Methods

### 4.1. Chemicals and Reagents

Pre-activated CNBr Sepharose 4B and HR 16/10 FPLC columns were purchased from GE Healthcare. Monoclonal Anti-GFPuv antibody and pGFPuv plasmid were purchased from Clontech. Fetuin glycoprotein from fetal bovine serum, β-Galactosidase enzyme, sialidase enzyme, goat IgG Anti-Mouse IgG-HRP conjugated secondary antibody, sintered glass filter (porosity G3) and all the chemicals (unless it is mentioned) were purchased from Sigma-Aldrich (Sigma-Aldrich, Dorset, UK).

### 4.2. Virtual Carbohydrate Docking

The crystal structures of MLI (2RG9) and SNA (3C9Z) were derived from RCSB Protein Data Bank as bdp files and they were converted to the final docking structures on Glycoprotein Builder tool on the online platform of Glycam project (www.glycam.org accessed on 3 February 2021). The structures of Neu5Ac and β-Galactose were drawn on ChemDraw software and converted to 3D structures on Carbohydrate Builder tool on Glycam project. The virtual carbohydrate docking was executed by using AutoDock tools.

### 4.3. Total RNA Extraction and cDNA Synthesis

Fresh leaves of European mistletoe plant were ground to a fine powder in liquid nitrogen by using mortar and pestle. The RNA extraction was performed by using ISOLATE II RNA plant kit (Meridian Bioscience, London, UK) following the manufacturer’s instructions. The quality of the extracted RNA was later assessed on agarose gel electrophoresis containing formaldehyde and NanoDrop2000 (Thermo Fisher scientific, Waltham, MA, USA). The cDNA was reverse transcribed using SuperScriptTM-II reverse transcriptase kit (Invitrogen, Waltham, MA, USA) following the manufacturer’s instructions.

### 4.4. Polymerase Chain Reactions (PCR)

The coding sequences of MLA and MLB chains of MLI were amplified by using reverse transcribed cDNA as a DNA template and Q5 High-Fidelity polymerase (NEB) with two sets of primer shown in Table 1.

### 4.5. Sequencing Reactions

The sequencing of the cloned MLA and MLB was carried out using BigDye Terminator cycle sequencing chemistries (Thermo Fisher scientific, USA) and standard forward and reverse M13 primers. The Prism 3100 Genetic Analyzer was used to run the capillary gels and the analysis of the sequencing data was carried out using SnapGene (SnapGene, San Diego, CA, USA), Chroma Lite and CLC Sequence Viewer (Qiagen, Hilden, Germany) software. The full-length sequences of SNA and MLI (MLA and MLB) from the sequencing reactions were aligned against the sequences of previously known glycan binding lectins.

### 4.6. Lectin-GFP Fusion Proteins

**Figure 9:**
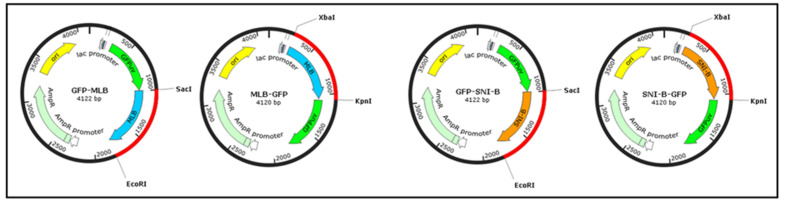
The cloning map of MLB and SNA-B N/C-terminally fused to green florescent protein (GFP) in pGFPuv plasmid.

Fusion proteins were generated from the binding chain (MLB) of both mistletoe lectin isoform one (MLI) and elderberry lectin isoform I (SNA-B). A reference gene of SNI-B from NCBI (U27122.1) was used as a template sequence for solid synthesis of the gene carried out by Eurofins Genomics (Eurofins, Luxembourg). The MLB and SNI-B sequences were then cloned in upstream and downstream to the coding sequence of GFP in pGFPuv plasmid (Figure 9).

### 4.7. On Plate Induction

The transformed *E. coli* cells with the plasmids containing the fusion protein constructs were plated on LB-Agar plates, which were surface-covered with 500 uM IPTG. The visualization and imaging of the grown colonies were performed under standard UV light (360–400 nm).

### 4.8. Expression of Recombinant MLB and SNA-B

The MLB + GFP and SNA-B + GFP fusion constructs were transformed into BL21(DE3) strain and plated on antibiotic selection plates (Ampicillin 100 μg/mL). The transformed cells were grown overnight in 10 mL of liquid LB with 100 μg/mL ampicillin at 37 °C. The overnight culture was added to a litter of fresh LB medium with antibiotic and incubated at 30 °C until OD600 0.7 was reached, followed by induction using 500 µM IPTG for 18 h. BugBuster protein extraction reagent was used to extract the total soluble proteins from the bacterial cells as per manufacture’s instruction. The extracted protein was later analyzed by SDS PAGE electrophoresis.

### 4.9. Affinity Column Preparation

Affinity column bed was prepared from the pre-activated CNBr Sepharose 4B which is an appropriate medium for immobilization of ligands containing primary amines. The medium was coupled with degalactosilated bovine fetuin as a heavily glycosylated protein containing bi-antennary, tri-antennary and tetra-antennary oligosaccharides with variable sialyation. Briefly, the required amount of lyophilized CNBr powder was weighed and suspended in 1 mM HCL. The medium swelled immediately and was washed on a sintered glass filter (porosity G3) with 1 mM HCl for 15 min. The required amount of fetuin (10 mg/mL) was also dissolved in dissolving buffer (0.1 M CH_3_COONa, pH 8.3; containing 0.5 M NaCl) and incubated at 37 °C overnight with an appropriate amount of β-galactosidase to remove the free galactose residues. The fetuin was then extensively dialyzed against a coupling buffer (0.1 M NaHCO3, pH 8.3; containing 0.5 M NaCl) and added to the suspended CNBr medium in a stoppered vessel. The mixture was subject to end-overend rotation overnight at 4 °C and washed later with 5 medium (gel) volumes of coupling buffer in order to wash away the excess ligands. The mixture was incubated in blocking buffer (0.1 M Tris-HCl) pH 8.0 for 2 h to block remaining active groups on Sepharose 4B and washed in three cycles of alternating pH with 5 medium volumes of 0.1 M acetic acid/sodium acetate, pH 4.0 containing 0.5 M NaCl, and 0.1M Tris-HCl, pH 8 containing 0.5 M NaCl. Finally, the coupled CNBr-fetuin bead was packed in HR 16/10 column.

### 4.10. Affinity Purification

Affinity column chromatography was performed in order to purify the fusion proteins from the total extracted proteins by using the pre-packed CNBr-fetuin column and AKTA prime FPLC platform. The column was equilibrated with running buffer (PBS, pH 6.0) and the effect of pH on the binding of MLB + GFP and SNA + GFP to the column was first analyzed through loading a small scale of MLB, SNA on the column and performing elution steps via three different pH (3.0, 6.0 and 9.0) of the elution buffer (PBS alone). The UV signal of the eluted fractions was monitored and compared to the elution step of a column loaded with only running buffer. At low pH points (3.0 and 6.0), none of the fusion proteins were observed to be eluted by the elution buffer and a negligible amount of the fusion proteins was eluted at pH 9.0. On the other hand, both fusion proteins were observed to be completely eluted from the column when sialic acid was added to the elution buffer at 6.0. Therefore, we decided to use pH 6.0 for the elution buffer, which was PBS containing 0.5 M sialic acid. Collected fractions containing the target fusion proteins were later analyzed on SDS-PAGE and Western blotting was used with the anti-GFPuv primary antibody.

### 4.11. Hemagglutination Assay

Erythrocytes were prepared by washing fresh sheep blood four times with 0.15 M NaCl and stored in Alsever’s medium (0.8% sodium citrate, 0.42% sodium chloride, 2.05% glucose and 0.055% citric acid) at 4 °C. The blood was centrifuged at 1500 *g* for 5 min to precipitate the erythrocytes, which was later washed with PBS four times in the ratio of 1:5 (*v*/*v*). A batch of the washed erythrocytes was treated with 10 unites/mL of sialidase enzyme for 2 h at 37 °C in order to remove the free sialic acid residues on their membranes and then used in the negative control treatment. Another batch of erythrocytes (4 mL) was added to 95 mL of PBS and 0.1% trypsin (*w*/*v*) and incubated for 90 min at 37 °C followed by four rounds of washing with PBS and diluting it to 1% erythrocytes suspension in PBS. Cells were incubated at 4 °C until use. The hemagglutination assay was performed on 96-well round bottom microtiter plates. In the first step, the minimum agglutination concentration (MAC) of MLB + GFP and SNA-B + GFP fusion proteins was identified by using a range of concentrations (0.05–1 µM) of the fusion proteins. For each well containing the fusion proteins 50 uL of 1% erythrocytes in PBS was added and incubated for two hours at room temperature. The agglutination of the erythrocytes was visually assessed. The identified MAC of both fusions proteins was then used in hemagglutination inhibition assay using a serial dilution of 1% erythrocytes. For the control treatments, the fusion proteins were pre-incubated with (10 mM) of three hemagglutination inhibitor agents (N-Acetylneuraminic acid (Neu5Ac), D-galactose (D-Gal) and D-glucose (D-Glc)) for an hour at 4 °C. The cells were added last and agglutination was permitted to proceed for 60 min at room temperature (22 °C).

### 4.12. Mammalian Cell Culture

Human metastatic melanoma cells (WM-266-4) (ECACC, Salisbury, UK) were cultured in DMEM medium supplemented with 1% (*v*/*v*) glutamine and 10% (*v*/*v*) fetal bovine serum (FBS) and maintained at 37 °C in a humidified atmosphere containing 5% carbon dioxide.

### 4.13. Luminescence Imaging Microscop

Human metastatic melanoma cells (WM 266-4) were plated on a 96-well plate at a seeding density of 8000 cells/well (~1.4 × 104 cells/cm^2^). Following overnight incubation, the FBS in the medium was lowered down to 2%. The cells were then treated with 0.3 µM of MLB-GFP and SNA-B-GFP fusion proteins and incubated for 18 h. The binding and internalization of the fusion proteins into the cells were visualized using luminescence imaging microscopy (Nikon, Tokyo, Japan).

## Figures and Tables

**Table 1:** Primers used in polymerase chain reaction, introduced restriction sites and added nucleotides shown in red; actual site underscored; main lectin sequence shown in black.

Primers	Sequences	TM (°C)
MLA-fwd	AGAGGAAGATGGCCGCTCTC	60
MLA-rev	TACGAGAGGCTAAGACTCAGAG	61
MLB-fwd	GATGATGTTACCTGCAGTGCTTC	60
MLB-rev	TGGCACGGGAAGCCACATTTG	61
MLB + SacI (fwd)	GGA GCT CGC TGC AGC TGC AGA TGA TGT TAC CTG	73
MLB + EcoRI (rev)	CGA ATT CAT TAT GCA GCT GGC ACG GGA AGC CAC	72
MLB + XbaI (fwd)	GCG TCT AGA AAA AGA TGA TGT TAC CTG CAG TGC TTC G	71
MLB + KpnI (rev)	GCA TGA GGT ACC TTT GGC ACG GGA AGC CAC	72
SNA-B + HindIII (fwd)	TGA AAG CTT G GGG GGC GAG TAC GAA AAA	66
SNA-B + XbaI (rev)	TGA TCT AGA GT AGC TGG ATG GGT GGT AGT	66

## Data Availability

Not applicable.

## Supplementary Materials

The following are available online at https://www.mdpi.com/article/10.3390/ijms22158284/s1.
